# Supplementary material for: Effectiveness of a community-based intervention package in maternal health service utilization: A cross-sectional quasi-experimental study in rural Ghana
Source: PLoS One. 2024 Nov 25;19(11):e0311966. doi: 10.1371/journal.pone.0311966 (PMC11588241; doi:10.1371/journal.pone.0311966)
Supplement: Attachment — Submitted filename: Comments.pdf [file pone.0311966.s001.pdf]

I am thankful for this opportunity of reading a very interesting research. I must emphasize that the manuscript will benefit immensely from a thorough editing to eliminate language-related and grammatical mistakes.

Abstract

Background

The main objective is not clearly written.

I would request the authors to consider writing something like this: "We examined the effectiveness of a community-based intervention package for pregnant women in increasing utilization of maternal health services. The package consisted of: (i)..., (ii)..., (iii)... and (iv) ...

Methods

I recommend the authors clarify how both baseline and follow-up data were collected simultaneously "after the implementation of the intervention package" (lines 102–104). How could baseline data be collected "*after* the implementation of the intervention package"? I found out the answer in lines 228–229, but readers should not be kept wondering about this. Thus, it needs to be clarified in the Abstract that by the word "simultaneously" the authors meant simultaneously in intervention and control sites.

The authors need to specify when the intervention was implemented (from X month to Y month of the year Z) in this section of the Abstract.

The authors need to mention the P-value that they considered for the level of statistical significance: $P < 0.05$ or a different one? I know that they have provided two reasons in lines 345–350, but here in the Abstract they need to mention that they considered $P < 0.10$ to indicate statistically significant associations.

Results

This section of the Abstract must be re-written. The authors need to present the answer to their main research question; i.e., the adjusted estimates for the three outcome variables.

I also recommend deleting lines 112–115 for the following two reasons:

i. whether the effectiveness of the intervention varied by categories of household wealth (assuming this to be a proxy for socio-economic status) was not the primary research question. In simpler words, whether household wealth or socio-economic status *modifies* the

“effect” of the intervention on the outcome was not the primary research question as per the explanations provided by the authors in lines 439–445;

ii. the authors kept comparing the stratum-specific odds ratio for women from the “poorer” households with the overall odds ratio for wealth level. Instead, the stratum specific odds ratio for women from the “poorer” households should be compared against stratum-specific odds ratios for the other two wealth categories; i.e., odds ratios for women from the “middle-income” and “richer” households. Moreover, it was not clear whether the authors tested the interaction term incorporating “group” and “wealth level” in the mixed-effects logistic regression model (the legend below Table 3, line 433 does not indicate so). Without the interaction term “group × level” being tested and its P-value being statistically significant, it would be erroneous to infer that household wealth or socio-economic status modifies the “effect” of the intervention on the outcome.

Conclusion

Lines 118–120 read, “Peer health education activities and training existing health workers are likely to have synchronously contributed to an increase in antenatal care coverage. This is largely because peer educators are friendlier to the women from poor households”. I strongly recommend the authors consider deleting these two sentences as they have done no analysis to support these inferences. These inferences regarding how or through which mechanism(s) did the intervention work were not examined by the authors in the study. Besides, I wonder how the authors would know: i. whether “peer health education” and training of “existing health workers” acted synchronously, and ii. peer educators were “friendlier to the women from poor households”?

I would recommend the authors reiterate the main result and comment on the public health relevance of the extent of increase in maternal health care utilization brought on by the intervention in the Conclusion. It would be insightful if the authors comment on whether they think the intervention should be scaled up based on their findings. I would assume the overall impact to be smaller than anticipated after an intervention that ran for more than 16 months (as per lines 238–243).

Main text

Background

Lines 158–161, please provide the reference for these interventions being effective at the end of the sentence.

Lines 161–165, please re-frame this long sentence containing 45 words.

Line 167, please omit the word “physical”.

The authors argued that home visits carried out by “formal health workers” do not lead to “expected effectiveness” as “formal health workers” are “not necessarily interactive and friendly enough to local women” (lines 174–177). I briefly looked into the paper that they cites as a reference for this argument (reference number 11, a study from Ghana by Konlan et al.), and could not find the part of the paper that supports the authors’ arguments. I would request the authors to help me find the part of the paper on which the authors based their argument. Otherwise, the authors need to find a different reference to support their argument.

Line 192, please omit the word “in”.

Lines 200–203 read “The Program also strengthened the functions and capacity of the existing community health management committees, to enable them to continuously support both CHVs and facility-based health workers”. The authors need to briefly mention how did the Program strengthen the “functions and capacity” of the committees.

Customarily, a reader would expect to find out the specific objective(s) of the study in the last paragraph of the Background. However, this was lacking in the manuscript. I would recommend the authors do this instead of what they wrote in lines 213–215.

Methods

I would strongly recommend the authors shed light on any theoretical framework or model and empirical evidence from the same or similar settings that they used to inform and design the intervention. Otherwise, the approach would appear to be “top down” and shaped by the funder’s agenda, not by the local community’s needs.

Under the subsection “Interventions and outcome variables”, the authors need to provide information regarding the educational status of the Maternal and Child Health Promoters and Peer Educators.

In lines 302–305, the authors need to describe how the “capacity of community health management committees was strengthened” for carrying out “evidence-based planning, implementation, monitoring and evaluation”. The authors avoided describing what was actually done “under the third pillar”. This leaves readers open to speculation.

Line 354, please provide a reference that outlines how principal component analysis is used for computing wealth index or asset scores.

In lines 356–358, the authors wrote that “Then, all the households were categorized into five groups (i.e. wealth quintiles) according to the values of wealth index”. However, in the Results section, in Figure 3 captioned “Economic-group-specific crude difference-in-difference for three outcome variables”, they appear to have collapsed the bottom two quintiles into one single category (labelled “Poorer”) and the top two quintiles into another single category (labelled “Richer”). This is a serious discrepancy in my opinion. I strongly recommend the authors either: i. present the DID for all five quintiles, or ii. they convert the values of wealth index into tertiles instead of quintiles and present the DID for all three tertiles.

It would be transparent, if the authors clearly mention which variables were considered random-effect and which were considered fixed-effect in the mixed-effects logistic regression.

I also suggest the authors mention how they assessed the adequacy or fit of the logistic regression models and how they checked for collinearity.

The authors wrote that they had access to attributes that could identify individual participants during and after data collection (lines 368–369). The authors need to mention under Ethical considerations how they have ensured the confidentiality of identifying attributes and personal information as well as what would happen to these in future. The authors need to mention whether participation was voluntary and whether the participants retained their right to withdraw throughout the study.

Results

It is a common practice in writing the Results section that only the findings are presented not any interpretation(s) of the finding(s). Nevertheless, the authors resorted to presenting interpretations and inferences in the Results section (lines 396–400 and 401–405). The authors need to remove these.

As mentioned above, the authors either: i. present the crude DID for all five quintiles, or ii. they convert the values of wealth index into tertiles instead of quintiles and present the DID for all three tertiles in Figure 3. Similarly, Table 4 should demonstrate the estimates for all five or three wealth groups, not just the estimates among “poorer” women. I also think both in Tables 3 and 4, the estimates for “Maternal age”, “Education attainment”, “Marital status”, “Enrolment in health insurance” and “Household size” should *not* be presented as the main research question does not deal with these variables. Not presenting these estimates would also make the tables less busy.

Discussion

In the first paragraph of this section, the authors need to interpret the difference in difference estimator in a way that would give the readers an impression about how much improvement was brought on by the intervention and what this means from an “effect size” perspective. Please see the comment for the Conclusion

Lines 473–475 read, “This indicates that ANC coverage significantly increased between pre- and post-intervention stages particularly among women from poor households in the intervention group”. I do not agree with how the sentence has been framed. The authors should consider writing something like this: out of the three aspects of maternal health service utilization, the intervention improved only the ANC visit coverage ... A positive impact of the intervention on ... ... was not observed.

Furthermore, I think the word “significantly” in line 473 is ambiguous: did the authors mean statistical significance? If so, they should write it clearly. Or, did the authors use “significantly” to indicate a considerably large “effect size”? In that case, the authors need to elaborate further.

I strongly recommend the authors remove what they wrote in lines 565–601. While it is very interesting to explore what might have happened when there were health systems disruptions from COVID-19, this should be reserved for another study. Moreover, the authors used the word “resilience”, and if I am to assume this to be a construct, then the question arises which variable captured this construct. I could not find any variable that systematically and validly captured resilience. In fact, the authors performed no specific analysis to support what they discussed in line 565–601; which makes it akin to speculation. The authors argued that “People’s overall hesitancy to go out should have made pregnant women in the control group refrain from making antenatal care visits. Despite generally increased hesitancy to go out, the

proportion of those having made at least four ANC visits slightly increased in the intervention group" (lines 585–589). There could be other confounding factors behind this small increase in proportions (unadjusted, bivariate estimate) of those completing at least four ANC visits among "poorer" women that were not possible to capture because of the study design.

I would prefer a Conclusion section where the authors present the principal finding in plain English and comment on the public health relevance of the extent of increase in maternal health care utilization brought on by the intervention and its scalability.
