# Supplementary material for: Effectiveness of a community-based intervention package in maternal health service utilization: A cross-sectional quasi-experimental study in rural Ghana
Source: PLoS One. 2024 Nov 25;19(11):e0311966. doi: 10.1371/journal.pone.0311966 (PMC11588241; doi:10.1371/journal.pone.0311966)
Supplement: Attachment — Submitted filename: Reviewers comments based on the document.docx [file pone.0311966.s002.docx]

Reviewers comments based on the document: “PONE-D-23-10669_revierer.pdf”

Manuscript

Title

Line 3: Effectiveness of a community-based intervention package (should there be an “a”)

Abstract

Line 93-97: When was this implemented?

Line 111-115: Could this be rephrased?

Background:

Line 154-155: Rephrase, what is meant with preventable causes? Maybe complications

Line 161-165: Not reader friendly, maybe: However poorer access to …..are major obstacles for the utilization of maternal health services. This prevents reproductive aged women to reach life-saving services

Line 170-171: What is meant with “intervening in women”?

Line 167-184: Is “cultivation” the right word to describe this? To me this does not what is aimed to say in the paragraph

Line: 186-204: Could this be put in a graph?

Methods

Line 237-253: Tell about the current health system, what services are offered to women and are the some groups that are less affected by the challenges explained in the intro or, on the other hand, especially vulnerable groups?

321: Sampling What were the exclusion criteria?

Line 345-348: Please clarify
