# Supplementary material for: Effectiveness of a community-based intervention package in maternal health service utilization: A cross-sectional quasi-experimental study in rural Ghana
Source: PLoS One. 2024 Nov 25;19(11):e0311966. doi: 10.1371/journal.pone.0311966 (PMC11588241; doi:10.1371/journal.pone.0311966)
Supplement: Attachment — Submitted filename: JOICFP&IMP-Jnl-PLoS ONE-Response-11Nov2023.docx [file pone.0311966.s003.docx]

**Responses to the reviewers’ COMMENTs**

We are extremely grateful to the editor and reviewers for their comments on the manuscript. We have significantly revised the manuscript based on those useful comments. Please refer to the following point-by-point-based responses to the respected comments, along with revised manuscript. Please note that the changes in the revised manuscript (i.e. main text, tables/figures, and reference list) have been shown in: **(i) red in this file for “*Responses to Reviewers*” as follows;** **(ii) track changes in the file “*Revised Manuscript with Track Changes*”;** and **(iii) red in the cleaned revised file “*Revised Manuscript*”**.

------------------------------------------------------

**COMMENTS FROM REVIEWER #1**

**[Comment 1-1]** I am thankful for this opportunity of reading a very interesting research. I must emphasize that the manuscript will benefit immensely from a thorough editing to eliminate language-related and grammatical mistakes.
**🡺 [Response 1-1]** We appreciate Reviewer #1 for his/her overall comments on this paper. We have addressed the specific comments and suggestions made by Reviewer #1 as follows.

**[Comment 1-2]** **Abstract/Background:** The main objective is not clearly written.
I would request the authors to consider writing something like this: “We examined the effectiveness of a community-based intervention package for pregnant women in increasing utilization of maternal health services. The package consisted of: (i)..., (ii)..., (iii)... and (iv) ...
**🡺 [Response 1-2]** Thanks for this concrete suggestion. The first sentence in ‘*Background*’ of Abstract has been revised according to this suggestion. Now, the manuscript reads “*We examined the effectiveness of a community-based intervention package that targeted pregnant women for increasing utilizations of maternal health services. The intervention package was…..*” (see lines 93-95 “*Revised Manuscript with Track Changes*”; lines 93-95 of “*Revised Manuscript*”).

**[Comment 1-3]** **Abstract/Methods:** I recommend the authors clarify how both baseline and follow-up data were collected simultaneously “after the implementation of the intervention package” (lines 102–104). How could baseline data be collected “after the implementation of the intervention package”? I found out the answer in lines 228–229, but readers should not be kept wondering about this. Thus, it needs to be clarified in the Abstract that by the word “simultaneously” the authors meant simultaneously in intervention and control sites.
**🡺 [Response 1-3]** This comment from Reviewer #1 is reasonable. The necessary revision has been made accordingly. Now, the manuscript reads “*A cross-sectional household survey was conducted in May 2022. We sampled four groups of women: (i) intervention at baseline; (ii) intervention at follow-up; (iii) control at baseline; and (iv) control at follow-up”.* (see lines 101-103 of “*Revised Manuscript with Track Changes*; lines 101-103 of “*Revised Manuscript*”). Please note that the word count limit for Abstract set by *PLoS One* (=300 words) does not allow us to write more details on how baseline and follow-up data were collected in a cross-sectional manner.

**[Comment 1-4]** **Abstract/Methods:** The authors need to specify when the intervention was implemented (from X month to Y month of the year Z) in this section of the Abstract.
**🡺 [Response 1-4]** To respond to this suggestion from Reviewer #1, an additional sentence “*The intervention package was implemented in Suhum Municipality, Ghana, from March 2019 to April 2022.”* has been inserted in the Background of Abstract (see lines 94-96 of “*Revised Manuscript with Track Changes*”; lines 94-96 of “*Revised Manuscript*”).

**[Comment 1-5]** **Abstract/Methods:** The authors need to mention the P-value that they considered for the level of statistical significance: P < 0.05 or a different one? I know that they have provided two reasons in lines 345–350, but here in the Abstract they need to mention that they considered P < 0.10 to indicate statistically significant associations.
**🡺 [Response 1-5]** Thanks for this careful comment. Based on the comment, a new sentence has been newly inserted in Methods of Abstract, accordingly, i.e. “*Significance levels were set at the values of 0.10, 0.05, and 0.01, since the aforementioned service coverages had been already too high to have room for an increase at the significance level of 0.05.*” (see lines 108-133 of “*Revised Manuscript with Track Changes*”; lines 108-110 of “*Revised Manuscript*”).

**[Comment 1-6]** **Abstract/Results:** This section of the Abstract must be re-written. The authors need to present the answer to their main research question; i.e., the adjusted estimates for the three outcome variables. I also recommend deleting lines 112–115 for the following two reasons: (i) whether the effectiveness of the intervention varied by categories of household wealth (assuming this to be a proxy for socio-economic status) was not the primary research question. In simpler words, whether household wealth or socio-economic status modifies the “effect” of the intervention on the outcome was not the primary research question as per the explanations provided by the authors in lines 439–445; (ii) the authors kept comparing the stratum-specific odds ratio for women from the “poorer” households with the overall odds ratio for wealth level. Instead, the stratum specific odds ratio for women from the “poorer” households should be compared against stratum-specific odds ratios for the other two wealth categories; i.e., odds ratios for women from the “middle-income” and “richer” households.

**🡺 [Response 1-6]** Thanks for this constructive comment. Having agreed to the reviewer’s suggestion, lines 112-115 in the previous manuscript have been deleted. Then, ‘*Results*’ of Abstract has been rewritten. Now, the manuscript reads “*The proportion of women having utilized at least four ANC services produced significant DID in both crude and adjusted estimates. The proportions of women having utilized facility-based delivery services and post-partum care services produced significant DID in neither crude nor adjusted estimates.*” (see lines 136-139 of “*Revised Manuscript with Track Changes*”; lines 113-116 of “*Revised Manuscript*”).

**[Comment 1-7]** **Abstract/Results:** Moreover, it was not clear whether the authors tested the interaction term incorporating “group” and “wealth level” in the mixed-effects logistic regression model (the legend below Table 3, line 433 does not indicate so). Without the interaction term “group × level” being tested and its P-value being statistically significant, it would be erroneous to infer that household wealth or socio-economic status modifies the “effect” of the intervention on the outcome.
**🡺 [Response 1-7]** Thanks for the comment. Table 3 was created for the purpose of showing overall effectiveness of the intervention package among all the respondents regardless of household’s wealth levels. In other words, it is not intended to show the comparison of the effectiveness between the poorer, middle-income, and richer. Therefore, it is not necessary to add the interaction terms of ‘*Group x Household’s wealth level*’ in the mixed-effect logistic regression models for Table 3. Thus, the way of analyzing data for Table 3 does not change. Please note that the categorization of ‘Household’s wealth groups was corrected based on the reviewer’s reasonable comment in **[Comment 1-20]**. However, the reviewer’s suggestion of inclusion of the interaction term has been usefully applied to calculation of wealth-level-specific DID in Table 4.

**[Comment 1-8]** **Abstract/Conclusion:** Lines 118–120 read, “Peer health education activities and training existing health workers are likely to have synchronously contributed to an increase in antenatal care coverage. This is largely because peer educators are friendlier to the women from poor households”. I strongly recommend the authors consider deleting these two sentences as they have done no analysis to support these inferences. These inferences regarding how or through which mechanism(s) did the intervention work were not examined by the authors in the study. Besides, I wonder how the authors would know: i. whether “peer health education” and training of “existing health workers” acted synchronously, and ii. peer educators were “friendlier to the women from poor households”?
I would recommend the authors reiterate the main result and comment on the public health relevance of the extent of increase in maternal health care utilization brought on by the intervention in the Conclusion. It would be insightful if the authors comment on whether they think the intervention should be scaled up based on their findings. I would assume the overall impact to be smaller than anticipated after an intervention that ran for more than 16 months (as per lines 238–243).
**🡺 [Response 1-8]** Based on this strong recommendation, ‘*Conclusions*’ of Abstract has been totally rewritten. Implication of the nationwide scaling-up of the intervention package has been added. Now, the manuscript reads “*Of the three outcome variables set, only the proportion of women having utilized at least four ANC services significantly increased in the intervention group, compared with the control group. Ghana has been in transition process of shifting the minimum number ANC visits from four to eight. Thus, nation-wide scaling up of the intervention package is expected to help the transition be smoothly completed though ensuring increasing in the number of ANC visits.*” (see lines 141-146 of “*Revised Manuscript with Track Changes*”; lines 119-124 of “*Revised Manuscript*”). We agree to the reviewer’s view that the interruption of the intervention package due to COVID-19 pandemic made the overall impact actually smaller and less significant than anticipated. Yet, the word count limit for Abstract (=300 words) does not allow us to write on this point. Hope the reviewer will kindly understand this.

**[Comment 1-9]** **Main text/Background:** Lines 158–161, please provide the reference for these interventions being effective at the end of the sentence.

**🡺 [Response 1-9]** To respond to this comment, three references have been added to the sentence “T*o reduce a significantly greater number of maternal deaths in Sub-Saharan Africa, timely and quality antenatal care (ANC) and facility-based delivery (FBD), post-partum care (PPC), Emergency Obstetric Care, and family planning are imperative.*” I.e. (i) Ref #5 - Sexual and Reproductive Health and Research, WHO. Maternal mortality: Evidence brief [Internet]. 2019. Available from: https://www.who.int/publications/i/item/WHO-RHR-19.20
(ii) Ref #6 - Paxton A, Maine D, Freedman L, Fry D, Lobis S. The evidence for emergency obstetric care. Intl J Gynecology & Obste. 2005 Feb;88(2):181–93.; and
(iii) Ref #7 - Levine R, Langer A, Birdsall N, Matheny G, Wright M, Bayer A. Contraception. In: Jamison DT, Breman JG, Measham AR, Alleyne G, Claeson M, Evans DB, et al., editors. Disease Control Priorities in Developing Countries [Internet]. 2nd ed. Washington (DC): The International Bank for Reconstruction and Development / The World Bank; 2006 [cited 2023 Oct 18]. Available from: http://www.ncbi.nlm.nih.gov/books/NBK11771/
(see line 213 and 907-918 of “*Revised Manuscript with Track Changes*”; line 164 and 672-683 of “*Revised Manuscript*”)

**[Comment 1-10]** **Main text/Background:** Lines 161–165, please re-frame this long sentence containing 45 words.
**🡺 [Response 1-10]** Thanks for the comment. The same/similar comment was raised by Reviewer #2 in **[Comment 2-7]**. Based on this reasonable comment from both reviewers, the sentence has been shortened, simplified and separated into two sentences. Accordingly, the number of words of the two sentences has been reduced from 45 to 32. Now, it reads **“***Poorer access to health facilities, inadequate quality of health services, and socio-economic/ cultural barriers are as the major obstacles in increasing maternal health service utilizations. This prevents reproductive-aged women from life-saving services*” (see lines 213-216 of “*Revised Manuscript with Track Changes*”; lines 164-167 of “*Revised Manuscript*”). See the **[Response 2-7]**, too.

**[Comment 1-11]** **Main text/Background:** Line 167, please omit the word “physical”.

**🡺 [Response 1-11]** Thanks for this suggestion. Having agreed to the reviewer’s suggestion, “physical” has been deleted. (see line 218 of “*Revised Manuscript with Track Changes*”; line 169 of “*Revised Manuscript*”).

**[Comment 1-12]** **Main text/Background:** The authors argued that home visits carried out by “formal health workers” do not lead to “expected effectiveness” as “formal health workers” are “not necessarily interactive and friendly enough to local women” (lines 174–177). I briefly looked into the paper that they cites as a reference for this argument (reference number 11, a study from Ghana by Konlan et al.), and could not find the part of the paper that supports the authors’ arguments. I would request the authors to help me find the part of the paper on which the authors based their argument. Otherwise, the authors need to find a different reference to support their argument.
**🡺 [Response 1-12]** Many thanks for carefully reviewing this part. We agree that the reference “*Konlan et al. 2021*” does nor clearly indicate poorer client-friendliness and interactivity of formal health workers’ demand creation (e.g. household visitis). The referencce “*Konlan D et al. 2021*” has been replaced by three new references, i.e. (i) Ref #15 – *Moyer CA, Adongo PB, Aborigo RA, Hodgson A, Engmann CM. ‘They treat you like you are not a human being’: Maltreatment during labour and delivery in rural northern Ghana. Midwifery. 2014 Feb;30(2):262–8.*; (ii) Ref #16 – *Dapaah JM, Nachinaab JO. Sociocultural Determinants of the Utilization of Maternal Health Care Services in the Tallensi District in the Upper East Region of Ghana. Advances in Public Health. 2019 Feb 10;2019:1–11.*; and (iii) Ref #17 – *Amu H, Nyarko SH. Satisfaction with Maternal Healthcare Services in the Ketu South Municipality, Ghana: A Qualitative Case Study. BioMed Research International. 2019 Apr 10;2019:1–6*.
(see line 243 and 953-963 of “*Revised Manuscript with Track Changes*”; line 179 and 713-723 of “*Revised Manuscript*”)

**[Comment 1-13]** **Main text/Background:** Line 192, please omit the word “in”.

**🡺 [Response 1-13]** Thanks for finding this typo. This “in” has been deleted, accordingly (see line 271 of “*Revised Manuscript with Track Changes*”; line 194 of “*Revised Manuscript*”).

**[Comment 1-14]** **Main text/Background:** Lines 200–203 read “The Program also strengthened the functions and capacity of the existing community health management committees, to enable them to continuously support both CHVs and facility-based health workers”. The authors need to briefly mention how did the Program strengthen the “functions and capacity” of the committees.

**🡺 [Response 1-14]** Thanks for this comment. An additional sentence has been newly inserted after this sentence “*Capacity strengthening of community health management committees was conducted through a series of lectures, groups works, and role plays.*” (see lines 282-284 of “*Revised Manuscript with Track Changes*”; lines 205-207 of “*Revised Manuscript*”).

**[Comment 1-15]** **Main text/Background:** Customarily, a reader would expect to find out the specific objective(s) of the study in the last paragraph of the Background. However, this was lacking in the manuscript. I would recommend the authors do this instead of what they wrote in lines 213–215.

**🡺 [Response 1-15]** We are thankful to the reviewer for this suggestion. An additional sentence “*Specific objectives of the study are to estimate the degree of increases in proportions of women having utilized: (i) at least four ANC visits; (ii) FBD; and (iii) PNC.*” has been inserted in the location the reviewer indicated (see lines 307-309 of “*Revised Manuscript with Track Changes*”; line 227-229 of “*Revised Manuscript*”).

**[Comment 1-16] Methods**: I would strongly recommend the authors shed light on any theoretical framework or model and empirical evidence from the same or similar settings that they used to inform and design the intervention. Otherwise, the approach would appear to be “top down” and shaped by the funder’s agenda, not by the local community’s needs.

**🡺 [Response 1-16]** Having agreed to this recommendation, three references have been newly added as model and empirical evidence for this intervention package, i.e. (i) Ref #29 – *UNFPA, Family Health International. Peer Education Toolkit [Internet]. 2006. Available from: https://www.unfpa.org/resources/peer-education-toolkit*; (ii) Ref #30 – *Japan Organization for International Cooperation in Family Planning (JOICFP). Improving Sexual Reproductive Health for the Youth in Tanzania [Internet]. 2018. Available from: https://www.joicfp.or.jp/eng/where_we_work/tanzania/*; and (iii) Ref #31 – *JOICFP. Maternal, Newborn and Child Health One Stop Service Project [Internet]. 2021. Available from: https://www.joicfp.or.jp/eng/where_we_work/zambia/maternal-newborn-and-child-health-one-stop-service-project/.*
(see line 325 and 1020-1029 of “*Revised Manuscript with Track Changes*”; line 242 and 769-777 of “*Revised Manuscript*”)

**[Comment 1-17] Methods**: Under the subsection “Interventions and outcome variables”, the authors need to provide information regarding the educational status of the Maternal and Child Health Promoters and Peer Educators.

**🡺 [Response 1-17]** Thanks for this suggestion. One sentence on education level of two types of CHVs (Maternal and Child Health Promoters and Peer Educators) has been inserted. “*As shown in Table 1, while a majority of Peer Educators completed either junior or senior high school education (81.3%), education attainments of Maternal and Child Health Promoters are more diverse.*” (see lines 397-400 of “*Revised Manuscript with Track Changes*”; lines 309-311 of “*Revised Manuscript*”). To show the detailed breakdown of education levels of Maternal and Child Health Promoters and Peer Educators “*Table 1 Education attainments of two types of community health volunteers*” has been newly added, too (see lines 402-403 of “*Revised Manuscript with Track Changes*”; lines 313-314 of “*Revised Manuscript*”). Accordingly, the numbering of subsequent tables has been adjusted. Also, as a typo in the number of Peer Educator (i.e. youth CHVs) was found, we corrected it accordingly, i.e. “*a total of 180 adult and 88 youth CHVs*” has been replaced by “*a total of 180 adult and 150 youth CHVs*” (see lines 396 of “*Revised Manuscript with Track Changes*”; lines 307 of “R*evised Manuscript*”).

**[Comment 1-18] Methods**: In lines 302–305, the authors need to describe how the “capacity of community health management committees was strengthened” for carrying out “evidence-based planning, implementation, monitoring and evaluation”. The authors avoided describing what was actually done “under the third pillar”. This leaves readers open to speculation.
**🡺 [Response 1-18]** Thanks for the comment similar to Comment 1-12. An additional sentence has been newly inserted after this sentence “*Capacity strengthening of community health management committees was conducted through a series of lectures, groups works, and role plays.*” (see lines 430-432 of “*Revised Manuscript with Track Changes*”; lines 336-338 of “*Revised Manuscript*”).

**[Comment 1-19] Methods**: Line 354, please provide a reference that outlines how principal component analysis is used for computing wealth index or asset scores.

**🡺 [Response 1-19]** Thanks for the comment. An additional sentence has been inserted, i.e. “*The principal component score was calculated for each household, by applying all the dichotomous variables of ownership of 11 types of utilities and assets to principle component analysis with varimax rotation.*” (see lines 499-502 of “*Revised Manuscript with Track Changes*”; lines 396-399 of “*Revised Manuscript*”). All the detailed processes of wealth index calculation cannot be described, at it will be unnecessarily lengthy. Thus, a reference has been added, i.e. (i) Ref #38 –Rutstein SO. Steps to constructing the new DHS wealth index. chrome-extension://efaidnbmnnnibpcajpcglclefindmkaj/https://dhsprogram.com/programming/wealth%20index/Steps_to_constructing_the_new_DHS_Wealth_Index.pdf. Accessed 21 September 2023.; (see line 502 and 1050-1053 of “*Revised Manuscript with Track Changes*”; line 399 and 798-801 of “*Revised Manuscript*”)

**[Comment 1-20]** In lines 356–358, the authors wrote that “Then, all the households were categorized into five groups (i.e. wealth quintiles) according to the values of wealth index”. However, in the Results section, in Figure 3 captioned “Economic-group-specific crude difference-in-difference for three outcome variables”, they appear to have collapsed the bottom two quintiles into one single category (labelled “Poorer”) and the top two quintiles into another single category (labelled “Richer”). This is a serious discrepancy in my opinion. I strongly recommend the authors either: i. present the DID for all five quintiles, or ii. they convert the values of wealth index into tertiles instead of quintiles and present the DID for all three tertiles.
**🡺 [Response 1-20]** Thanks for this reasonable comment. Since the sample size is not great enough for five-group comparison, we employed three-group comparison. Now we used tertile for three-group comparison, by agreeing to your suggestion. Accordingly, several parts of the manuscript have been revised, as follows. First, “*into* ***five*** *groups (i.e. wealth* ***quintiles****).*” was replaced by “*into* ***three*** *groups (i.e. wealth* ***tertiles****).*” (see lines 503 of “*Revised Manuscript with Track Changes*”; lines 400 of “*Revised Manuscript*”). Table 5 (former Table 4) now presents an inter-wealth-group comparison of DID for all three tertiles (see lines 654 of “*Revised Manuscript with Track Changes*”; lines 504 of “*Revised Manuscript*”). The final paragraph of the Results section has been revised, accordingly, i.e. “*Table 5 shows the results of mixed-effect logistic regressions for the three outcome variables with an interaction between DID estimates and wealth levels. Women from poor households in the intervention group exhibited a significant increase in the likelihood of utilizing ANC services, compared with those in the control group (aOR = 2.63; P = 0.059). Also, our analysis did not reveal a variation in the effectiveness of the intervention package for the other outcomes across wealth tertiles.*” (see lines 624-629 of “*Revised Manuscript with Track Changes*”; lines 497-502 of “*Revised Manuscript*”).

**[Comment 1-21] Methods**: It would be transparent, if the authors clearly mention which variables were considered random-effect and which were considered fixed-effect in the mixed-effects logistic regression.
**🡺 [Response 1-21]** Many thanks for your suggestion. Having agreed to the suggestion, we inserted the phrase “*by applying sub-district as the random effect of and independent variables (i.e. group, time, maternal age, education attainment, marital status, enrollment in health insurance, household size, and household’s wealth level) as the fixed effects.*” (see lines 479-482 of “*Revised Manuscript with Track Changes*”; lines 379-382 of “*Revised Manuscript*”).

**[Comment 1-22] Methods**: I also suggest the authors mention how they assessed the adequacy or fit of the logistic regression models and how they checked for collinearity.
**🡺 [Response 1-22]** Many thanks for your suggestion. Having agreed to the suggestion, we inserted the phrase “*To test possible multicollinearity, variance inﬂation factor (VIF) was assessed for each independent variable, by applying VIF value equal to or greater than 10 as presence of multi-collinearity*” (see lines 482-484 of “*Revised Manuscript with Track Changes*”; lines 382-384 of “*Revised Manuscript*”). As the results of this way of checking multicollinearity, none of independent variables produced VIF≥10. Thus, two sentences have been inserted in the Results section “*None of independent variables produced VIF values equal to or greater than 10. Thus, no multicollinearity was assumed.*” (see lines 586-588 of “*Revised Manuscript with Track Changes*”; lines 467-469 of “*Revised Manuscript*”).

**[Comment 1-23] Methods**: The authors wrote that they had access to attributes that could identify individual participants during and after data collection (lines 368–369). The authors need to mention under Ethical considerations how they have ensured the confidentiality of identifying attributes and personal information as well as what would happen to these in future. The authors need to mention whether participation was voluntary and whether the participants retained their right to withdraw throughout the study.
**🡺 [Response 1-23]** Thanks for your suggestion. Having agreed to the suggestion, we inserted the phrase “*To ensure confidentiality of personal data of the participants, the dataset for analyses was anonymized by encoding the data with unique participant identification numbers. Another dataset composed exclusively of the identification numbers and participants’ personal data (i.e. name and phone numbers) was separately created. Both datasets were protected by using the different passwords.*” (see lines 518-522 of “*Revised Manuscript with Track Changes*”; lines 413-417 of “*Revised Manuscript*”).

**[Comment 1-24] Results**: It is a common practice in writing the Results section that only the findings are presented not any interpretation(s) of the finding(s). Nevertheless, the authors resorted to presenting interpretations and inferences in the Results section (lines 396–400 and 401–405). The authors need to remove these.

**🡺 [Response 1-24]** Thanks. Based on this comment, we deleted the sentence “*Thus, the respondent mothers in both groups were equally educated enough to be able to be health-literate and contactable enough to be adequately informed of any upcoming individual peer education sessions and other health-related events, which are key elements of the Program.*” and phrase “……*who are more likely to flexibly accept new lifestyles (incl. sexual and reproductive behaviors)[26,27]. Thus, both groups are expected to be more likely to be sensitive to and curious about newly introduced approaches that were delivered and shared by peer health education and promotion through individual and group health education sessions*” (see lines 552 of “*Revised Manuscript with Track Changes*”; lines 448 of “*Revised Manuscript*”).

**[Comment 1-24] Results**: As mentioned above, the authors either: i. present the crude DID for all five quintiles, or ii. they convert the values of wealth index into tertiles instead of quintiles and present the DID for all three tertiles in Figure 3. Similarly, Table 4 should demonstrate the estimates for all five or three wealth groups, not just the estimates among “poorer” women. I also think both in Tables 3 and 4, the estimates for “Maternal age”, “Education attainment”, “Marital status”, “Enrolment in health insurance” and “Household size” should not be presented as the main research question does not deal with these variables. Not presenting these estimates would also make the tables less busy.
**🡺 [Response 1-24]** As previously addressed in **[Response 1-20]**, the texts for description of Table 5 has been revised, accordingly, i.e. tertile has been used instead of quintiles for Table 4 (former Table 3), Table 5 (former Table 4), and Figure 3. Now, Table 5 (former Table 4) indicates inter-wealth group comparisons of effectiveness of the intervention, by showing the difference in DID estimates between the wealth groups. We respect the reviewer’s recommendation of deletion of “Maternal age”, “Education attainment”, “Marital status”, “Enrolment in health insurance” and “Household size” in Table 4 (former Table 3) and Table 5 (former Table 4). Yet, we clearly mentioned that “*This indicates that a need for calculating an adjusted DID estimate for each outcome variable by controlling its confounders, in addition to estimating its crude DID*” in Results section (see lines 548-550 of “*Revised Manuscript with Track Changes*”; lines 444-446 of “*Revised Manuscript*”). Therefore, we maintain those variables in the tables, to clearly show those confounder variables have been controlled. Otherwise, the audience would misunderstand as if those confounders had not been addressed. The reviewers are kindly requested to fully understand and consider our views and concerns. Moreover, we understand the reviewer’s view of need for removals of estimates of independent variables from Table 4 (former Table 3) and Table 5 (former Table 4), to clearly streamlining the contents. We have given careful consideration to the reviewer’s recommendation. Instead of removing estimates, we would like to emphasize the importance of retaining all the results in Table 4 (former Table 3) and Table 5 (former Table 4) for comprehensive reporting. We believe that taking a balance between the primary focus and comprehensive reporting enhances the utility and versatility of our research findings. However, if the reviewer strongly recommends the removals of esimates, please kindly let us know, and we will proceed accordingly.

**[Comment 1-25] Discussion**: In the first paragraph of this section, the authors need to interpret the difference in difference estimator in a way that would give the readers an impression about how much improvement was brought on by the intervention and what this means from an “effect size” perspective. Please see the comment for the Conclusion

**🡺 [Response 1-25]** Thanks for this suggestion. The first paragraph of Discussion section has been revised accordingly, i.e. “*Of the three types of essential maternal health service utilizations (ANC, FBD and PPC), the intervention package improved only ANC visit coverage. A positive impact of the intervention package on FBD and PPC was not observed. Moreover, an adjusted DID estimate for ANC among women from poor households was significantly positive (aOR = 2.23; P = 0.059 <0.10).*” (see lines 667-671 of “*Revised Manuscript with Track Changes*”; lines 511-515 of “*Revised Manuscript*”).

**[Comment 1-26] Discussion**: Lines 473–475 read, “This indicates that ANC coverage significantly increased between pre- and post-intervention stages particularly among women from poor households in the intervention group”. I do not agree with how the sentence has been framed. The authors should consider writing something like this: out of the three aspects of maternal health service utilization, the intervention improved only the ANC visit coverage ... A positive impact of the intervention on ... ... ... was not observed.
**🡺 [Response 1-26]** Thanks for this suggestion. Having agreed to the suggestion, the first sentence of the first paragraph of Discussion section reads “*Of the three types of essential maternal health service utilizations (ANC, FBD and PPC), the intervention package improved only ANC visit coverage. A positive impact of the intervention package on FBD and PPC was not observed.*” (see lines 667-669 of “*Revised Manuscript with Track Changes*”; lines 511-513 of “*Revised Manuscript*”).

**[Comment 1-27] Discussion**: Furthermore, I think the word “significantly” in line 473 is ambiguous: did the authors mean statistical significance? If so, they should write it clearly. Or, did the authors use “significantly” to indicate a considerably large “effect size”? In that case, the authors need to elaborate further.
**🡺 [Response 1-27]** Thanks for the clarification. To clearly address the ambiguity, we have added aOR value along with its P-value “*Moreover, an adjusted DID estimate for ANC among women from poor households was significantly positive (aOR = 2.23; P = 0.059 <0.10).*” (see lines 669-671 of “*Revised Manuscript with Track Changes*”; lines 513-515 of “*Revised Manuscript*”).

**[Comment 1-28] Discussion**: I strongly recommend the authors remove what they wrote in lines 565–601. While it is very interesting to explore what might have happened when there were health systems disruptions from COVID-19, this should be reserved for another study. Moreover, the authors used the word “resilience”, and if I am to assume this to be a construct, then the question arises which variable captured this construct. I could not find any variable that systematically and validly captured resilience. In fact, the authors performed no specific analysis to support what they discussed in line 565–601; which makes it akin to speculation. The authors argued that “People’s overall hesitancy to go out should have made pregnant women in the control group refrain from making antenatal care visits. Despite generally increased hesitancy to go out, the proportion of those having made at least four ANC visits slightly increased in the intervention group” (lines 585–589). There could be other confounding factors behind this small increase in proportions (unadjusted, bivariate estimate) of those completing at least four ANC visits among “poorer” women that were not possible to capture because of the study design.
**🡺 [Response 1-28]** Thanks for the comment. Having agreed to this comment, lines 565-601 have been deleted. (see lines 790 of “*Revised Manuscript with Track Changes*”; lines 608 of “*Revised Manuscript*”).

**[Comment 1-29] Conclusion**: I would prefer a Conclusion section where the authors present the principal finding in plain English and comment on the public health relevance of the extent of increase in maternal health care utilization brought on by the intervention and its scalability.

**🡺 [Response 1-29]** We appreciate this comment. ‘Conclusion’ section has been added, accordingly. I.e. “Conclusion. *Of the three outcome variables set, only the proportion of women having utilized at least four ANC services significantly increased in the intervention group, compared with the control group. We recommend the intervention package be scaled up either nationwide or selectively to the areas where greater room for improvement in ANC coverage is identified. Ghana has been in transition process of shifting the minimum number ANC visits from four to eight. This indicates the additional need for an overall nationwide increase in the number of ANC visits. Thus, scaling up of the intervention package tested in this study is expected to help the transition be smoothly completed though ensuring increasing in the number of ANC visits*.” (see lines 869-877 of “*Revised Manuscript with Track Changes*”; lines 634-642 of “*Revised Manuscript*”).

**COMMENTS FROM REVIEWER #2**

**[Comment 2-1]** The study is on an important subject with precious information on interventions and women's health.

**🡺 [Response 2-1]** We are thankful to the reviewer for recognizing the importance of this study.

**[Comment 2-2]** Regarding the abstract, it could be revised to be more on point and send a clearer message.

**🡺 [Response 2-2]** Thanks for the suggestion. Based on the comments of need for clarity and accuracy of Abstract from both Reviewer #1 and Reviewer #2, Abstract has been revised, accordingly (see lines 91-146 of “*Revised Manuscript with Track Changes*”; lines 91-124 of “*Revised Manuscript*”).

**[Comment 2-3] Comment 1 in the attachment. Title:** Line 3: Effectiveness of a community-based intervention package (should there be an “a”)

**🡺 [Response 2-3]** Thanks for the suggestion. Having agreed to this reviewer’s view, the study title has been revised. Now it is “*Effectiveness of* ***a*** *community-based intervention package in maternal health service utilizations: A cross-sectional quasi-experimental study in rural Ghana*” (see lines 3-5 of “*Revised Manuscript with Track Changes*”; lines 3-5 of “*Revised Manuscript*”).

**[Comment 2-4] Comment 2 in the attachment: Abstract:** Line 93-97: When was this implemented?

**🡺 [Response 2-4]** Thanks for the suggestion. Having agreed to this reviewer’s view, the study title has been revised. The implementation period has been inserted, i.e. “*The intervention package was implemented in Suhum Municipality, Ghana,* ***from March 2019 to April 2022.***” (see lines 94-96 of “*Revised Manuscript with Track Changes*”; lines 94-96 of “*Revised Manuscript*”)

**[Comment 2-5] Comment 3 in the attachment: Abstract:** Line 111-115: Could this be rephrased?

**🡺 [Response 2-5]** Thanks for the comment. This is a common comment between Reviewer #1 and Reviewer #2. Abstract/Results has been revised, accordingly, by makng it more straightforward and simpler, i.e. “*The proportion of women having utilized at least four ANC services produced significant DID in both crude and adjusted estimates. The proportions of women having utilized facility-based delivery services and post-partum care services produced significant DID in neither crude nor adjusted estimates.*” (see lines 136-139 of “*Revised Manuscript with Track Changes*”; lines 113-116 of “*Revised Manuscript*”).

**[Comment 2-6] Comment 4 in the attachment: Background:** Line 154-155: Rephrase, what is meant with preventable causes? Maybe complications

**🡺 [Response 2-6]** Thanks for the comment. The initial part of Background section has been enriched, accordingly. Now it reads “*Today, approximately 800 women die due to the preventable causes of maternal deaths in the world, every day. Preventable causes of maternal deaths are composed of direct causes (obstetric hemorrhage, hypertensive disorders, puerperal sepsis, and unsafe abortion) and indirect causes (HIV and AIDS, malaria, tuberculosis, other sexually transmitted infections, malnutrition). They can be prevented through increasing availability of and access to quality of sexual, reproductive, maternal care.*” (see lines 201-207 of “*Revised Manuscript with Track Changes*”; lines 152-158 of “*Revised Manuscript*”).

**[Comment 2-7] Comment 5 in the attachment: Background:** Line 161-165: Not reader friendly, maybe: However poorer access to …..are major obstacles for the utilization of maternal health services. This prevents reproductive aged women to reach life-saving services.

**🡺 [Response 2-7]** Thanks for the comment. The same/similar comment was raised by Reviewer #2 in **[Comment 1-10]**. Based on this reasonable comment from both reviewers, the sentence has been shortened, simplified and separated into two sentences. Accordingly, the number of words of the two sentences has been reduced from 45 to 32. Now, it reads **“***Poorer access to health facilities, inadequate quality of health services, and socio-economic/ cultural barriers are as the major obstacles in increasing maternal health service utilizations. This prevents reproductive-aged women from life-saving services*” (see lines 213-216 of “*Revised Manuscript with Track Changes*”; lines 164-167 of “*Revised Manuscript*”). See the **[Response 1-10]**, too.

**[Comment 2-8] Comment 6 in the attachment: Background:** Line 170-171: What is meant with “intervening in women”?

**🡺 [Response 2-8]** Thanks for clarification. Since it seems that “intervening in women” is not clear enough, the phrase has been reworded. Now, the phrase “*…by directly intervening in women in communities…*” has been replaced by “*…through direct intervention on women in communities…*”(see lines 236-237 of “*Revised Manuscript with Track Changes*”; lines 172-173 of “*Revised Manuscript*”).

**[Comment 2-9] Comment 7 in the attachment: Background:** Line 167-184: Is “cultivation” the right word to describe this? To me this does not what is aimed to say in the paragraph

**🡺 [Response 2-9]** “Demand cultivation” is often used. Yet, the term has been replaced by “demand creation”, as it does not make sense to Reviewer #2. (see lines 236 of “*Revised Manuscript with Track Changes*”; lines 172 of “*Revised Manuscript*”; lines 238 of “*Revised Manuscript with Track Changes*”; lines 174 of “*Revised Manuscript*”; lines 240 of “*Revised Manuscript with Track Changes*”; lines 176 of “*Revised Manuscript*” ; lines 716 of “*Revised Manuscript with Track Changes*”; lines 547 of “*Revised Manuscript*”; lines 718 of “*Revised Manuscript with Track Changes*”; lines 549 of “*Revised Manuscript*”)

**[Comment 2-10] Comment 8 in the attachment: Background:** Line: 186-204: Could this be put in a graph?

**🡺 [Response 2-10]** Thanks for this suggestion. Yet, there is no data in this paragraph (line 186-204 in the previous version). Thus, we don’t understand what graph, Reviewer #2 is expecting. Moreover, it is not recommended usually that a graph or table be included in Background section. Therefore, no specific change has been made, base on this comment.

**[Comment 2-11] Comment 9 in the attachment: Methods:** Line 237-253: Tell about the current health system, what services are offered to women and are the some groups that are less affected by the challenges explained in the intro or, on the other hand, especially vulnerable groups?

**🡺 [Response 2-11]** Thanks for the comment. While fully understanding this comment, detailed types of services to be provided through CHPS initiative has been now described in Background section in responding to **[Comment 2-15]** (see **🡺 [Response 2-15]** below). Moreover, repeatedly describing the types of services available in Methods section is not very appropriate. Therefore, a sentence “*In both Suhum Municipality (intervention site) and Atiwa West District (control site), all the basic health services described earlier were made available.*” has been inserted (see lines 362-364 of “*Revised Manuscript with Track Changes*”; lines 273-275 of “*Revised Manuscript*”). In addition, to specify the type of vulnerable group who are largely affected by challenges in accessing health services, three sentences have been inserted into Background section (= Introduction section), i.e. “*National Health Insurance Scheme (NHIS) covers the significant part of maternal and child health service costs for those insured. Yet, not all costs are covered by NHIS and, needless to say, out-of-pocket payments made by those not insured become much larger. Thus, the poor have greater challenges in utilizations of health services.*” (see lines 309-313 of “*Revised Manuscript with Track Changes*”; lines 229-233 of “*Revised Manuscript*”).

**[Comment 2-12] Comment 10 in the attachment: Methods:** Line 321: Sampling What were the exclusion criteria?

**🡺 [Response 2-12]** Thanks for clarification on exclusion criteria. Now two sentences have been inserted in Methods section. I.e. “*Those having delivered during the period other than the above two periods were excluded from the sampling. Moreover, those having been relocated, absent despite three repeated visits or refused to participate in the study were excluded, too.*” (see lines 458-461 of “*Revised Manuscript with Track Changes*”; lines 360-363 of “*Revised Manuscript*”).

**[Comment 2-13] Comment 11 in the attachment: Methods:** Line 345-348: Please clarify

**🡺 [Response 2-13]** Thanks for clarification. Yet, we strongly believe that this part “*Significance levels were set at the values of 0.10, 0.05, and 0.01. The reason for unusually employing 0.10 as a significance level was that the service coverages of the outcome variables had been already too high to have room for an increase at the significance level of 0.05 (e.g. 93.1% of ANC in the intervention group at baseline).*” Is clear and precise enough. In fact, Reviewer #1 clearly understood and recognized this part as the reasonable significance level setting (see lines 484-491 of “*Revised Manuscript with Track Changes*”; lines 384-388 of “*Revised Manuscript*”).

**[Comment 2-14]** Additionally as it is based on an intervention, I would recommend to make a graphical depiction on how the intervention was carried out.

**🡺 [Response 2-14]** Thanks for the suggestion. Having agreed to develop a graphic depiction for how intervention package was implemented, “*Figure 2 Design of the Intervention package*” has been newly created and inserted. (see lines 394 of “*Revised Manuscript with Track Changes*”; lines 305 of “*Revised Manuscript*”). Accordingly, the numbering of subsequent figures has been adjusted.

**[Comment 2-15]** This study might be read, and used also in other contexts and thus it is important to include context specific information on current healthcare services for women of reproductive age.

**🡺 [Response 2-15]** Thanks for the suggestion. The description of Ghana-context-specific information on maternal and child health services has been inserted in the final paragraph of Background section, i.e. “*In Ghana, the Community-based Health Planning and Services (CHPS) initiative has been implemented since 2000 after careful piloting [28]. The initiative is aimed at ensuring availability of and access to basic maternal and child health services (antenatal care, delivery, postpartum care, child immunization, growth monitoring, and integrated management of childhood illnesses), in particular consideration to women in rural areas. Two community health officers are assigned at each CHPS compound, a primary health facility, to enable them to provide both facility-based and community-based maternal and child health*” (see lines 299-307 of “*Revised Manuscript with Track Changes*”; lines 219-227 of “*Revised Manuscript*”).

END
