# Supplementary material for: Effectiveness of a community-based intervention package in maternal health service utilization: A cross-sectional quasi-experimental study in rural Ghana
Source: PLoS One. 2024 Nov 25;19(11):e0311966. doi: 10.1371/journal.pone.0311966 (PMC11588241; doi:10.1371/journal.pone.0311966)
Supplement: Attachment — Submitted filename: JOICFP&IMP-Jnl-PLoS ONE-Response-R3.docx [file pone.0311966.s004.docx]

**Responses to the reviewers’ COMMENTs**

We are extremely grateful to the editor and reviewers for their comments on the manuscript. We have revised the manuscript based on the useful comments. Please refer to the following point-by-point-based responses to the respective comments, along with revised manuscript. Please note that the changes in the revised manuscript (i.e. main text, tables/figures, and reference list) have been shown in: **(i) red in this file for “*Responses to Reviewers*” as follows;** **(ii) track changes in the file “*Revised Manuscript with Track Changes*”;** and **(iii) red in the cleaned revised file “*Revised Manuscript*”**.

------------------------------------------------------

**COMMENTS FROM REVIEWER #3**

**[Comment 3-1]** The authors addressed most of the comments made in the previous round of peer review appropriately. Please consider the following remaining comments:
**🡺 [Response 3-1]** Thanks for recognizing the responses and revision we have made for the previous version.

**[Comment 3-2]** 1. Design effect: In the responses-to-reviewers document, the authors explained that the design effect (1.3) used for sample size calculations was based on the Ghana DHS 2022 Report. However, the data collection started on May 2022, before the data collection of the Ghana DHS 2022 (starting on October 2022), the authors might not access to the information in the report when the survey was designed. In addition, it would be noted that 1) design effect is a function of Intraclass Correlation Coefficient (ICC) and cluster size (for example, https://doi.org/10.1016/j.jeph.2024.202198) and 2) cluster size may be different between DHS and this study as it depends on the sampling design of a study. Therefore, it is possible that the design effect expected in this study was larger than the design effect in DHS (if the [average] cluster size of this study is larger than the cluster size of DHS). It is unclear about the cluster size in this study (it should be reported if it is not mentioned in the Methods section), and the cluster size (or the number of samples in the second stage) of Ghana DHS 2022 was 30 according to its report (https://www.dhsprogram.com/pubs/pdf/FR387/FR387.pdf). Thus, if the average cluster size of this study was smaller than Ghana DHS, the authors’ sample size calculation might be sufficient (even if they knew it after the DHS report was issued). Otherwise, the authors might want to add explanations on their initial sample size calculations more in details in the Methods section and a possibility of a smaller sample size than it should have been as a limitation of this study in the Discussion section.
**🡺 [Response 3-2]** Thanks for sharing the detailed insights on design effect and two-stage sampling. As *Reviewer #3* assumed, the design effect of 1.3 reported in *Ghana DHS 2022 Report* was accessed only after our data collection was completed. That was why this point and its reference (i.e. *Ghana DHS 2022 Report*) were not inserted into the main text when revising the manuscript last time. In view of this comment from *Reviewer #3*, the phrase “***where mean cluster size was 25***” has been inserted into the main text (see Line 373 in revised manuscript with TC; and Line 369 in revised manuscript without TC). We have reservation about adding more explanations on the sample size calculation in relation to design effect, for the three reasons: (i) the number of words of the main text has already great enough (i.e. 5,030) though *PLoS One* does not specify the word count limit; (ii) this study is not intended to focus on the detailed on sampling technique; and (iii) this manuscript has been submitted to *PLoS One* which is the journal not specialized in the journal specialized in epidemiology and/or biostatistics.

**[Comment 3-3]** 2. Percentage points: In Figures 2 and 3, the authors added the explanation that “Crude DID is expressed in form of percentage difference.” However, the DID may be captured as a difference between the percentages. For example, in the panel (a) of Figure 2, Crude DID was 4.6 percentage points (= [93.4% – 93.1%] – [92.4% – 96.7%] = 0.3% – [-4.3%]). Therefore, in the panel (a), it may be appropriate to present “Crude DID = 4.6 percentage points” instead of “Crude DID = 4.6%.” (If this is correct, the authors might want to revise similar presentations in Figures 2 and 3.
**🡺 [Response 3-3]** Thanks for suggesting this correction. Accordingly, the phrase “*Crude DID = X.XX percentage points*” has been used for each figure in Figure 3 and Figure 4. Moreover, “*Crude DID is expressed in form of percentage* ***difference***” has been replaced by “*Crude DID is expressed in form of percentage* ***points***” (the footnotes for both Figure 3 and Figure 4 in the revised manuscript with TC and revised manuscript without TC).

**[Comment 3-4]** 3. Percentage points: In relation to the comment above, the explanation of the Results section might be re-examined. For example, in Lines 487-, the authors explained that “Again, ANC was the only outcome variable that produced significant crude DID (11.5%; P = 0.082 < 0.10).” This percentage presentation (11.5%) might also be 11.5 percentage points. In addition, if it referred to the result presented in Figure 4 [1a] (ANC for women from poor 33%), it would be 11.4 percentage points and p = 0.085, according to the figure. Please check if the explanation in the main text was consistent with what was presented in the figure.
**🡺 [Response 3-4]** Many thanks for suggesting this correction in line with the above **[Comment 3-3]**. Accordingly, “……*significant crude DID (11.5 %; P = 0.082)*.” has been replaced by “……*significant crude DID (11.4 percentage points; P = 0.085)*.” (see Line 500-501 in revised manuscript with TC; and Line 495-496 in revised manuscript without TC)

**[Comment 3-5]** Line 123-124: please consider writing "*... the transition be smooth by increasing the number of ANC visits.*"
**🡺 [Response 3-5]** Thanks for identifying this typo by carefully reviewing the revised manuscript. Accordingly, the phrase "*... the transition be smooth by* ***increasing in the*** *number of ANC visits.*" has been replaced by "*... the transition be smooth by* ***increasing the number*** *of ANC visits.*" (see Line 122-123 in revised manuscript with TC; and Line 122-123 in revised manuscript without TC).

-----------------------------------------------------------------------------

**COMMENTS FROM REVIEWER #4**

**[Comment 4-1]** Introduction 1. “This is largely because they are not necessarily interactive and friendly enough to local women” line 178. Who is this sentence referring to?
**🡺 [Response 4-1]** Thanks for this clarification. Accordingly, “*This is largely because* ***they are*** *not necessarily interactive and friendly enough to local women*” has been replaced by “*This is largely because* ***the formal health workers' way of demand creation is not*** *necessarily interactive and friendly enough to local women*” (see Line 177 in revised manuscript with TC; and Line 177 in revised manuscript without TC). Hope this will help *Reviewer #4* and the audience of the article will more clearly understand this part.

**[Comment 4-2]** Introduction 2. “Peer education has been often employed” – line 180 – grammar??
**🡺 [Response 4-2]** Thanks for this clarification. This way of using the verb “*employ*” is common. For example, “*The peer education program has been* ***employed*** *as one of the strategies for HIV prevention*” (Hayashi M, Evaluation of the Peer Education Program in the Central Region of Vietnam. *Journal of the National Institute of Public Health*. 2009; **58** (4): 408) and “*Peer education interventions are commonly* ***employed*** *to prevent HIV*” (Abdi F, Simbar M. The Peer Education Approach in Adolescents- Narrative Review Article. [*Iran J Public Health*](https://www.ncbi.nlm.nih.gov/pmc/journals/1793/). 2013; **42** (11): 1200-1206). Many other earlier publications used “*employ*” in this manner. Moreover, in fact, none of the three other reviewers for our manuscript raised this clarification. This implies the sentence “*Peer education has been often employed*” is well understood. Thus, no change has been made in the manuscript.

**[Comment 4-3]** Introduction 3. “a study needs to be conducted in Ghana”. Line 216 and 217 – this sentence should be re-stated. The study is done now, apparently.
**🡺 [Response 4-3]** Thanks for this suggestion. We agreed to make a necessary change in this sentence. Now, “… ***a study needs to be*** *conducted in Ghana*” has been replaced by “… ***this study was*** *conducted in Ghana*” (see Line 220 in revised manuscript with TC; and Line 217 in revised manuscript without TC).

**[Comment 4-4]** Introduction 4. “This is because the implementation of the Program was less affected by the pandemic of SARS-CoV-2 (COVID-19) pandemic in Ghana, compared with other three countries.” Line 218 – why? Unless this can be explained with evidence, the statement is more or less a conjecture and should be revised.
**🡺 [Response 4-4]** Thanks for this clarification. There is some evidence for the phrase “… *the Program was less affected by the SARS-CoV-2 (COVID-19) pandemic in Ghana*” compared with three other countries. First, the Ghanaian government established partial lockdowns in COVID-19 hotspots, primarily in Greater Accra Region and Greater Kumasi Region on 30^th^ March 2020. Thus, Eastern Region in which both Suhum Municipality (intervention group) and Atiwa West District (control group), the target areas of this study, were not included in the partial lockdown areas. Second, Ghana was one of the first African countries that lifted the lockdowns ([Knott S. Ghana’s decision to lift partial COVID lockdown criticized by some. *VOA News*. April 20, 2020.](Knott%20S.%20Ghana’s%20decision%20to%20lift%20partial%20COVID%20lockdown%20criticized%20by%20some%20(VOA%20News.%20April%2020,%202020.%20Accessed%20February%206,2023. ) <https://www.voanews.com/a/africa_ghanas-decision-lift-partial-covid-19-lockdown-criticized-some/6187869.html>).

**[Comment 4-5]** Introduction 5. at least four ANC visits; (ii) FBD; and (iii) PNC. National Health Insurance Scheme (NHIS) line 218. Why use abbreviation in stating your objectives?
**🡺 [Response 4-5]** Thanks for clarification. First, “*PNC*” was the typo of “*PPC*”. Second, the three abbreviations “*ANC*”, “*FBD*” and “*PPC*” have appeared before this part in Line 160-162. I.e. “*To reduce a significantly greater number of maternal deaths in Sub-Saharan Africa, timely and quality antenatal care (ANC) and facility-based delivery (FBD), post-partum care (PPC)* ….” (see Line 160-162 in revised manuscript with TC; and Line 160-162 in revised manuscript without TC). Fully spelling out an abbreviation should be done only once, when it appears for the first time. Thus, no change has been made in Line 235-236.

**[Comment 4-6]** Methods 1. “Ghana was targeted for the study. i.e. Group I1 as the intervention group at baseline, Group I2 as the intervention group at follow-up, Group C1 as the control group at baseline, and Group as the control group at follow-up (Figure 1)”. – line 253. It does not clear to me whether baseline and follow up groups for both intervention and control had same of member for the time difference. Otherwise, how did you manage the difference in characteristics if each group had different participants?
**🡺 [Response 4-6]** Thanks for this clarification. Needless to say, the women who were pregnant at the time of the baseline (from 1^st^ January 2018 to 28^th^ February 2019) could be generally expected not to be pregnant again at the time of the follow-up (from 1^st^ August 2021 to 30^th^ April 2022), too. Thus, the study participants in baseline and follow-up groups were different. Yet, there were a few possibilities that some women having been pregnant at the time of both baseline and follow-up. For this reason, we systematically checked the presence of those women. At the result, it was found that no women who were pregnant at the time of both baseline and follow-up were selected for any of the four groups. Please refer to the two sentences “*All the women who delivered between 1^st^ January 2018 and 28^th^ February 2019 before the Program’s launch in March 2019 were included in the baseline sampling frames for both intervention and control groups (Group I_1_ and Group C_1_ in Figure 1). Similarly, all the women who delivered between 1^st^ August 2021 and 30^th^ April 2022 after the 10 months had passed since the Program’s launch were included in the follow-up sampling frames for both intervention and control groups (Group I_2_ and Group C_2_ in Figure 1)*.*”* (see Line 289-295 in revised manuscript with TC; and Line 282-288 in the revised manuscript without TC).

**[Comment 4-7]** Methods 2. “Atiwa West District was appropriate as the control group also because its socio-economic and socio-demographic characteristics, and maternal health service coverages at the baseline were reportedly at the similar level to those of Suhum Municipality.” – line 269, 270 – Atiwa West district is close to Suhum. They share border. How did you manage spillover effect?? If you did not, state it as a limitation.
**🡺 [Response 4-7]** Thanks for this clarification. The possibilities of contamination of a series of interventions into Atiwa West District (control group) must have been limited for the two reasons. First, Atiwa West District (control group) is closely located to Suhum Municipality (intervention group) but without sharing their boarder (see the map below). Since the total number of tables and figures is too many (i.e. 9 = 5 tables and 4 figures), we decided not to add this map to the revised manuscript.

**Atiwa West District**

**Suhum Municipality**

Second, during COVID-19 pandemic, people’s mobility and internal migration within Ghana were regulated and refrained from. For example, the graph below (<https://www.exemplars.health/emerging-topics/ecr/ghana/how-did-ghana-respond>) shows the reduction in people’s movements during the COVID-19 pandemic. Nevertheless, we cannot negate some possibilities of contamination. Therefore, an additional paragraph has been inserted into the final part of Discussion section. I.e. “*Fourth, there are limited possibilities of contamination of a series of interventions into the control group. This is because: (i) Atiwa West District (control group) and Suhum Municipality (intervention group) are not mutually adjacent; and (ii) people’s movement was regulated and voluntarily refrained from during COVID-19 pandemic. Yet, a certain level of the possibilities of contamination cannot be negated.*” (see Line 648-652 in revised manuscript with TC; and Line 638-642 in the revised manuscript without TC).

**[Comment 4-8]** Design and intervention 1. Ghana and indeed the WHO had abandoned 4 plus visit, even before 2018. What was the motivation for setting your outcome variable at 4+ instead of 8+?
Results
**🡺 [Response 4-8]** Thanks for raising this point. As *Reviewer #4* mentioned, Ghana Health Service (GHS) recommended all pregnant women make at least eight ANC visits in *Ghana National Safe Motherhood Protocol 2017*. Yet, in view of this unrealistic and infeasible target of eight or more NC visits, GHS relaxed the target figure for the minimum number of ANC visits from eight back to four in 2020. In fact, *Ghana National Safe Motherhood Protocol 2021* employs at least four ANC visits as the minimum number of ANC visits (GHS, *Ghana National Safe Motherhood Protocol 2021*. Accra: GHS; 2021. page 3) 4+ANC has been serving as the only monitoring indicator of frequency of ANC visits in GHS (GHS, *Health Information Management System. Standard Operating Procedure 4^th^ edition 2020*. Accra: GHS; 2017. page 337). Even, *Ghana DHS 2022* employed exclusively at least four ANC visits by excluding at least eight ANC visits as the variable, through respecting this GHS’s relaxing the national standard (Ghana Statistical Service, *Ghana Demographic and Health Survey 2022*. Accra: Ghana Statistical Service; 2022. Page 158.) To avoid unnecessary complicated explanation which is not essential to this study, we decided not to insert any additional sentences and phrases on this topic into the main text.

**[Comment 4-9]** final stage of the five-year Program – line 433. Authors may take this sentence away from results section and move it to methods section.
**🡺 [Response 4-9]** Thanks for this suggestion. The phrase “*, the final stage of the five-year Program*” has been simply deleted without moving it to Methods section. This is because this phrase is essential neither in Results section nor in Methods section. (see Line 445 in revised manuscript with TC; and Line 438 in revised manuscript without TC).

**[Comment 4-10]** Discussion: “*Of the three types of essential maternal health service utilizations (ANC, FBD and PPC), the intervention package improved only ANC visit coverage. A positive impact of the intervention package on FBD and PPC was not observed*” – line 507 to 509 – it appears to me that analysing with 8+ plus in accordance WHO current recommendation even before this intervention was rolled out would have given different results altogether. Authors can re-run with 8+ or offer strong explanation to support their choice of 4+
**🡺 [Response 4-10]** Thanks for this suggestion of further analysis on 8+ANC visits as an additional dependent variable. While agreeing to *Reviewer #4*’s view that this additional analysis may provide some useful insights on the feasibility of 8+ANC in Ghana, we would like not to do so. There are two reasons for it. First, as described in **[Response 4-8]**, Ghana Health Service now officially has adopted and recommends 4+ANC visits as the only monitoring indicator for ANC visit frequency (see GHS, *Ghana National Safe Motherhood Protocol 2021*. Accra: GHS; 2021. page 3). By respecting this policy of Ghana Health Service, *Ghana Demographic and Health Survey 2022* also reported 4+ANC visits as the only monitoring indicator for ANC visit frequency (see Ghana Statistical Service, *Ghana Demographic and Health Survey 2022*. Accra: Ghana Statistical Service; 2022. Page 158.), too. Second, there are already three dependent variables (= outcome indicators) (i.e. 4+ANC, FBD, PPC). Having the fourth dependent variable (= outcome indicator) as an additional one makes the paper look complicated.

**[Comment 4-11]** Overall, a clean statistical analysis.

**🡺 [Response 4-11]** Thanks for this comment.

END
